# Asciminib in Philadelphia Chromosome-Positive Acute Lymphoblastic Leukemia: A Case Series and Review of Emerging Evidence

**DOI:** 10.3390/hematolrep18020028

**Published:** 2026-04-13

**Authors:** Mostafa F. Mohammed Saleh, Abdulrahman Nasiri, Ahmed Kotb Abdrabou, Hadeel Samarkandi, Ayman Saad, Mahmoud Aljurf, Amr Hanbali, Ali Alahmari

**Affiliations:** 1 Adult Hematology, Transplantation and Cellular Therapy Department, Oncology Center, King Faisal Specialist Hospital and Research Center, Riyadh 11211, Saudi Arabia; 2 Clinical Hematology Unit, Department of Internal Medicine, Faculty of Medicine, Assiut University, Assiut 71515, Egypt; 3 College of Medicine, Imam Mohammad Ibn Saud Islamic University, Riyadh 13318, Saudi Arabia; 4 Clinical Hematology Unit, Internal Medicine Department, Faculty of Medicine, Zagazig University, Zagazig 44519, Egypt; 5 Department of Pharmacy, Cancer Centre of Excellence, King Faisal Specialist Hospital and Research Center, Riyadh 11211, Saudi Arabia

**Keywords:** Philadelphia chromosome-positive acute lymphoblastic leukemia, asciminib, BCR, ABL1 inhibition, relapsed/refractory disease

## Abstract

Philadelphia chromosome-positive acute lymphoblastic leukemia (Ph+ ALL) remains a high-risk entity despite advances in tyrosine kinase inhibitors (TKIs), immunotherapy, and cellular therapies. Relapse driven by clonal evolution, central nervous system (CNS) sanctuary disease, and TKI resistance, particularly T315I mutations, continues to limit durable disease control. Asciminib, a first-in-class allosteric *BCR::ABL1* (STAMP) inhibitor, has demonstrated efficacy and favorable tolerability in chronic myeloid leukemia, but its optimal role in Ph+ ALL remains to be defined. We report a three-patient case series of Ph+ acute leukemia treated with asciminib across diverse high-risk clinical settings, including multiply relapsed disease, CNS involvement, T315I-mutated leukemia, post-CAR-T-cell relapses, and transplant bridging. Clinical outcomes are contextualized through a comprehensive review of emerging clinical trial data, real-world cohorts, and mechanistic studies evaluating asciminib in Ph+ ALL. Across all cases, asciminib was incorporated as part of combination or consolidation strategies rather than as monotherapy in active disease. Asciminib contributed to molecular disease control, CNS leukemia clearance, and successful bridging to allogeneic transplantation or cellular therapy, with acceptable tolerability and no major vascular toxicity. Integration of published evidence demonstrates that asciminib exhibits consistent biological activity in Ph+ ALL, with improved durability when used in rational combinations, particularly with immunotherapy or ATP-competitive TKIs. Preclinical data further support asciminib’s compatibility with antibody-based and cellular therapies through preservation of immune effector function. Asciminib represents a versatile but context-dependent therapeutic option in Ph+ ALL. Its greatest clinical value appears to lie in rational combination regimens, maintenance strategies, and bridging to definitive therapies rather than single-agent salvage. Emerging structural biomarkers and ongoing clinical trials are expected to further refine patient selection, sequencing, and optimal integration of asciminib, particularly in CNS-involved disease and post-CAR-T cell relapse.

## 1. Introduction

Philadelphia chromosome-positive acute lymphoblastic leukemia (Ph+ ALL) is characterized by the *BCR::ABL1* fusion oncogene, which constitutively activates downstream kinases (SRC, STAT5, PI3K/AKT, RAS/RAF/MEK), accounting for approximately 20–30% of adult ALL cases [1,2]. Ph+ B-ALL can arise either de novo from committed B-cell precursors or represent a lymphoid blast crisis emerging from the transformation of underlying chronic myeloid leukemia (CML) [3]. While de novo Ph+ ALL and CML lymphoid blast crisis share overlapping clinical presentations and are driven by the same core molecular pathogenesis, they exhibit distinct genomic and prognostic profiles; de novo disease is typically characterized by the p190 *BCR::ABL1* transcript and a slightly more favorable prognosis, whereas CML transformation more commonly features the p210 transcript and additional cytogenetic abnormalities indicative of progressive clonal evolution [3,4]. Standard initial treatments often overlap, relying on intensive TKI-based combinations, but long-term outcomes and the biological interpretation of measurable residual disease (MRD) differ between the two entities [3].

The therapeutic landscape has been fundamentally transformed over the past two decades through the integration of tyrosine kinase inhibitors (TKIs) into chemotherapy backbones and has shifted the paradigm from a uniformly fatal diagnosis to one where long-term survival is increasingly achievable [5,6]. However, despite the successes of first- and second-generation agents, the disease remains characterized by a relentless capacity for clonal evolution and resistance; relapsed or refractory disease remains common, particularly in patients with advanced disease, central nervous system (CNS) involvement, prior exposure to multiple TKIs, or high-risk kinase domain mutations such as T315I [7]. The development of the third-generation TKI, ponatinib, marked a significant milestone, particularly for its ability to overcome the “gatekeeper” T315I mutation [8]. Nevertheless, the clinical utility of ponatinib is frequently circumscribed by its dose-dependent cardiovascular toxicity, leaving a critical therapeutic void for patients who are intolerant to this agent or who relapse despite its use. The landscape has further expanded with antibody-based immunotherapy and CD19-directed chimeric antigen receptor T-cell (CAR-T) therapy; however, outcomes after relapse following these modalities remain poor, and optimal sequencing and maintenance strategies are undefined [7].

Asciminib is a first-in-class allosteric inhibitor that specifically targets the ABL1 myristoyl pocket (STAMP inhibitor), restoring the inactive conformation of *BCR::ABL1*. This unique mechanism distinguishes asciminib from ATP-competitive TKIs and results in a non-overlapping resistance profile and improved selectivity [9]. While asciminib has demonstrated robust efficacy and tolerability in chronic myeloid leukemia, its role in Ph+ ALL is still evolving. Early-phase trials, real-world cohorts, and case reports suggest that asciminib may be particularly valuable when used in combination regimens, as a bridge to cellular therapy or transplantation, and as maintenance therapy in high-risk settings [10].

Here, we report three complex cases of Ph+ acute leukemia treated with asciminib and discuss these experiences in the context of emerging clinical and biological evidence.

## 2. Case Descriptions

**Case 1**: A 29-year-old female was diagnosed in May 2018 with Philadelphia chromosome-positive chronic myeloid leukemia, presenting as lymphoblastic blast crisis with CD19-positive disease. She received induction therapy with Hyper-CVAD cycle 1A combined with dasatinib but demonstrated refractory disease on day-22 bone marrow evaluation. Salvage chemotherapy with FLAG-IDA plus dasatinib achieved disease control, allowing progression to allogeneic hematopoietic stem cell transplantation (allo-HSCT) using cyclophosphamide and total body irradiation conditioning in August 2018. Post-transplant relapse was treated with two cycles of blinatumomab (administered with an extended interval due to infectious complications) between December 2018 and February 2020. Due to persistent high-risk disease, a second allo-HSCT using fludarabine/melphalan conditioning was performed in April 2020. In April 2021, the patient relapsed with combined CNS involvement and measurable residual disease (MRD)-positive marrow and subsequently received lymphodepleting chemotherapy followed by tisagenlecleucel CD19-directed CAR-T therapy in July 2021, achieving a durable remission. Molecular relapse was detected in March 2024, and ponatinib 15 mg daily was initiated. In January 2025, she presented with a severe headache and rising *BCR::ABL1* transcript levels (0.6%), with MRD-positive marrow and suspected CNS disease, precluding lumbar puncture. Kinase domain mutation analysis was negative. She was treated with blinatumomab re-induction combined with intrathecal therapy, achieving complete molecular remission, MRD-negative marrow, and radiographic CNS response. However, treatment was complicated by immune effector cell-associated neurotoxicity syndrome and a further isolated CNS relapse in July 2025, necessitating placement of an Ommaya reservoir. Given repeated CNS relapses and prior intolerance or resistance to multiple ATP-competitive TKIs, asciminib was introduced and escalated to 200 mg twice daily as systemic disease-controlling and CNS-bridging therapy. This high-dose strategy (200 mg twice daily) was adopted based on pharmacological modeling and clinical trial data demonstrating safety and enhanced efficacy against resistant clones in advanced Ph+ leukemias [11]. Formal therapeutic drug monitoring (TDM) was not performed. Following asciminib dose escalation in combination with regular intrathecal chemotherapy, cerebrospinal fluid examinations demonstrated clearance of blasts, and *BCR::ABL1* transcripts became and remained undetectable by December 2025. Asciminib was well tolerated, and no severe adverse events attributable to the drug were observed, specifically without any vascular, pancreatic, or severe hematologic toxicity. The patient remains clinically stable on asciminib maintenance with ongoing intrathecal therapy and is being evaluated for repeat CD19-directed CAR-T therapy.

**Case 2**: A 37-year-old male with chronic myeloid leukemia diagnosed in 2018 (chronic phase) was treated sequentially with imatinib, with suboptimal response, followed by dasatinib, achieving major molecular response before treatment discontinuation and loss to follow-up. In January 2024, he relapsed with Philadelphia chromosome-positive pre-B acute lymphoblastic leukemia in blast crisis, harboring a *BCR::ABL1* T315I mutation with cerebrospinal fluid involvement. He was refractory to blinatumomab and ponatinib but achieved marrow MRD negativity after two cycles of inotuzumab ozogamicin, with clearance of CNS disease following intrathecal thiotepa. He subsequently underwent brexucabtagene autoleucel CAR-T therapy in January 2025 after cyclophosphamide/fludarabine lymphodepletion, achieving MRD-negative marrow and complete metabolic response. His post-CAR-T course was complicated by grade 1 cytokine release syndrome and severe immune-mediated polyradiculopathy, requiring intravenous immunoglobulin and corticosteroids. Given high-risk molecular features, asciminib 40 mg twice daily was initiated in February 2025 as post-CAR-T disease control. Molecular relapse occurred by July 2025 with overt lymphoid blast crisis and re-emergence of T315I-mutated *BCR::ABL1* (>55%). He received induction with mini-CVAD, inotuzumab, and blinatumomab, alongside escalation of asciminib to 200 mg twice daily, with transient ponatinib re-challenge. He achieved complete remission with MRD at the detection limit and proceeded to matched sibling donor allo-HSCT in October 2025 using TBI/etoposide-based conditioning. Post-transplant, he achieved timely neutrophil and platelet engraftment, with manageable mucositis and no severe graft-versus-host disease. At the last follow-up in December 2025, he remains in molecular remission with undetectable *BCR::ABL1*, maintained on asciminib 200 mg twice daily. Similar to Case 1, the high dose of asciminib was safely administered without the occurrence of any severe adverse events.

**Case 3**: A 30-year-old male with Philadelphia chromosome-positive (p190) B-cell acute lymphoblastic leukemia was diagnosed in November 2019 and achieved complete remission following hyper-CVAD plus dasatinib, followed by matched sibling allo-HSCT using cyclophosphamide and total body irradiation conditioning in March 2020. His disease course was characterized by multiple relapses, including a peripheral blood relapse in April 2021 responding to ponatinib-based therapy, a molecular relapse in August 2024 managed with ponatinib, and a major relapse in November 2024 complicated by acute coronary syndrome. This relapse was treated with inotuzumab ozogamicin and high-dose asciminib, achieving MRD-negative remission by December 2024. In June 2025, he experienced a fourth relapse with circulating blasts (~35%) and bone marrow involvement (~25%). Ponatinib 30 mg daily and asciminib 40 mg twice daily were initiated with the intent to bridge to CAR-T therapy; however, this plan was deferred due to rapid clinical deterioration. He was admitted with a central line-associated bloodstream infection and developed severe hypoxemic respiratory failure consistent with acute respiratory distress syndrome, complicated by septic shock, stage III acute kidney injury requiring continuous renal replacement therapy, profound pancytopenia with gastrointestinal bleeding, and suspected invasive fungal pneumonia. Bronchoalveolar lavage grew *Candida tropicalis*, and he received broad-spectrum antibacterial and antifungal therapy. His ICU course was further complicated by seizure-like episodes with MRI findings suggestive of rhombencephalitis. Despite maximal ventilatory, vasopressor, renal, and antimicrobial support, he developed refractory multiorgan failure. In accordance with a previously established do-not-attempt-resuscitation order, no cardiopulmonary resuscitation was performed, and he died from cardiorespiratory arrest secondary to refractory hypoxemic respiratory failure on 24 July 2025. Asciminib and ponatinib were held during critical illness.

Table 1 summarizes the clinical characteristics and treatment course of three patients with Philadelphia chromosome-positive acute lymphoblastic leukemia treated with asciminib.

## 3. Discussion

This three-case series illustrates the expanding but context-dependent role of asciminib in Ph+ ALL, encompassing multiply relapsed disease, CNS involvement, T315I-mutated leukemia, post-CAR-T cell relapse, and transplant bridging. When integrated with emerging clinical, real-world, and mechanistic evidence, these observations support a convergent paradigm in which asciminib demonstrates consistent biological activity across heterogeneous Ph+ ALL settings. However, durable clinical benefit appears contingent on rational combination strategies, appropriate disease timing, and biologically informed patient selection, rather than reliance on asciminib monotherapy.

In contrast to CML, where large phase 3 randomized trials such as ASCEMBL and ASC4FIRST have established asciminib as a standard-of-care option, the evidence base supporting its use in Ph+ ALL remains limited and is derived primarily from early-phase clinical trials, compassionate use programs, and retrospective real-world analyses. Consequently, available data must be interpreted with caution, with explicit acknowledgment of the selection bias and confounding inherent to non-randomized cohorts [10,12].

Initial proof-of-concept for asciminib in Ph+ ALL derives from the first-in-human ALL-724 phase I study, which demonstrated measurable antileukemic activity in heavily pretreated patients, including those with T315I and compound ABL1 mutations [13]. Early molecular stabilization was observed in more than half of evaluable patients, with *BCR::ABL1* ≤ 0.1% achieved in approximately 50% by week 4. However, responses were short-lived, and most patients discontinued therapy due to disease progression, establishing that asciminib monotherapy, while biologically active, is insufficient for durable disease control in advanced Ph+ ALL.

These findings align with our experience. In none of the three cases was asciminib used as stand-alone therapy in active disease. Instead, asciminib was consistently introduced after cytoreduction or in combination regimens, underscoring that disease burden and biological complexity critically determine clinical benefit.

The earliest clinical signal supporting asciminib combination therapy came from the seminal report by Zerbit et al., describing durable disease control exceeding 17 months with combined asciminib and ponatinib in a heavily pretreated patient with relapsed/refractory Ph+ ALL [14]. This report provided the first clinical validation of dual allosteric and ATP-competitive *BCR::ABL1* inhibition, supported by preclinical synergy data. Subsequent reports have consistently reinforced this strategy. Amin Muhammad et al. described effective asciminib–ponatinib use as a bridge to brexucabtagene autoleucel CAR-T-cell therapy and as post-CAR-T maintenance, achieving sustained molecular remission without prohibitive toxicity [15]. Similarly, our Case 2 illustrates successful escalation of asciminib to 200 mg twice daily alongside transient ponatinib re-challenge, enabling disease control and subsequent allogeneic transplantation in T315I-mutated Ph+ ALL.

A critical element of asciminib’s integration into the therapeutic algorithm is the use of “rational combination strategies.” This concept refers to combining asciminib with agents that have complementary mechanisms of action or non-overlapping resistance profiles. Key examples include dual kinase inhibition (combining the allosteric STAMP inhibitor asciminib with an ATP-competitive TKI like ponatinib to prevent mutational escape) and chemo-immunotherapy combinations (pairing asciminib with blinatumomab or inotuzumab ozogamicin to provide deep molecular control while leveraging distinct immunological mechanisms of cell death) [14,16].

Real-world data further support this approach. In the multicenter French cohort of 41 patients with relapsed/refractory Ph+ ALL or CML lymphoid blast crisis, asciminib achieved an overall hematologic response rate of 83%, with MRD negativity in 57% of responders [17]. Importantly, event-free survival was significantly longer when asciminib was used in combination rather than as monotherapy, strongly favoring combination-based deployment.

Multiple reports support the compatibility of asciminib with antibody-based therapies. Rapid and deep MRD-negative remissions have been reported with asciminib combined with inotuzumab ozogamicin in elderly, multiply relapsed patients who were ineligible for intensive chemotherapy [16]. In addition, successful bridging to allogeneic transplantation has been described using asciminib at 200 mg twice daily in combination with mini-hyper-CVD and inotuzumab, with molecular remission achieved in most patients [18]. The available clinical studies and published case reports evaluating asciminib in Ph+ ALL are summarized in Table 2.

The safety profile of asciminib represents a major advantage over older-generation TKIs, but it is not devoid of toxicities. Real-world and clinical trial data indicate that the most common adverse effects include fatigue, thrombocytopenia, and anemia. Unlike ponatinib, asciminib has a significantly lower risk of severe cardiovascular and occlusive arterial events [20]. However, notable severe (grade 3/4) adverse events can occur, including pancreatitis (characterized by elevated lipase levels), hypertension, and profound myelosuppression. Cross-toxicity with prior TKIs has been observed for cytopenias and pancreatitis, emphasizing the need for careful monitoring, particularly when high doses (200 mg twice daily) are utilized in heavily pretreated populations [20,21].

These clinical observations are reinforced by preclinical immunologic data. In contrast to ATP-competitive TKIs such as dasatinib and ponatinib, asciminib preserves natural killer cell-mediated antibody-dependent cellular cytotoxicity and macrophage phagocytosis when combined with anti-CD20 monoclonal antibodies [22]. Independent studies have confirmed maintenance of ADCC across multiple B-ALL models with asciminib, whereas ATP-competitive TKIs markedly impair immune effector function [23]. This distinct immunologic profile provides a strong biological rationale for combining asciminib with immune-based therapies, including blinatumomab, inotuzumab, rituximab, and CAR-T cell therapy, consistent with the clinical strategies employed across all three cases.

The use of asciminib following CAR-T therapy, as illustrated in Cases 1 and 2, is particularly compelling. The rationale for post-CAR-T asciminib maintenance lies in its ability to exert continuous allosteric suppression of *BCR::ABL1* clones without causing the profound T-cell immunosuppression associated with dasatinib or ponatinib, theoretically allowing for prolonged persistence and function of CAR-T-cells [24]. Regarding timing in the salvage setting, asciminib is best introduced either as an early bridge to cellular therapy when disease burden is low, or immediately post-infusion/post-transplant as maintenance, rather than waiting for overt morphologic relapse where its single-agent efficacy is rapidly overcome [17].

Our cases also highlight the importance of tracking biological and molecular evidence during therapy. In Case 1, we observed a direct correlation between asciminib dose escalation and the clearance of *BCR::ABL1* transcripts from the CNS. In Case 2, the rapid re-emergence of the T315I clone following CAR-T therapy necessitated immediate high-dose asciminib intervention, demonstrating that while asciminib can control resistant clones, mutational dynamics must be closely monitored.

The distinction between de novo Ph+ ALL and CML lymphoid blast crisis (LBC) is also highly relevant to asciminib’s efficacy. While both entities are driven by *BCR::ABL1*, de novo Ph+ ALL predominantly expresses the p190 transcript, which generates a fusion protein with stronger kinase activity and a distinct signaling network compared to the p210 transcript typical of CML [19]. Despite these fundamental molecular differences, recent real-world data suggest that asciminib achieves comparable hematologic response rates and MRD negativity in both de novo Ph+ ALL and CML-LBC when used in the relapsed/refractory setting, implying that its allosteric mechanism effectively inhibits both major transcript variants [17].

The phase I trial by Luskin et al. represents a critical advance by demonstrating the feasibility of incorporating asciminib earlier in therapy. In newly diagnosed Ph+ ALL and lymphoid blast crisis, asciminib combined with dasatinib and prednisone achieved rapid and deep responses, with 100% complete hematologic remission by day 84 and MRD negativity by flow cytometry in 89% of patients [19]. Molecular responses were also substantial, with 74% achieving *BCR::ABL1* < 0.1%. These results suggest that asciminib may enhance the depth of response and potentially mitigate resistance when introduced earlier, supporting ongoing trials evaluating asciminib-based frontline and chemotherapy-free regimens. Ongoing and planned clinical trials evaluating asciminib across frontline, salvage, and maintenance settings in Ph+ ALL are summarized in Table 3.

Beyond cytoreduction, asciminib appears to exert disease-modifying effects at the stem cell level. Chatain et al. demonstrated that asciminib monotherapy suppressed aggressive lymphoid blast crisis in vivo, selectively depleted leukemic stem cells, restored normal hematopoiesis, and prevented disease propagation in secondary transplantation models [25]. These findings provide a biological explanation for asciminib’s effectiveness as a maintenance or consolidation agent, particularly in MRD-positive states. This concept is reinforced by the Haematologica Spotlight Review by Hughes et al., which positions asciminib as a paradigm-shifting therapy whose optimal utility in Ph+ ALL lies in combination strategies, maintenance therapy, and relapse prevention rather than single-agent salvage [26].

Recent mechanistic studies have refined patient selection for asciminib. Eadie et al. demonstrated that asciminib efficacy against ABL1- and ABL2-rearranged ALL is critically dependent on retention of the ABL SH3 domain, with SH3-deficient fusions showing primary resistance despite an intact myristoyl pocket [27]. Complementary work by Lagonik et al. extended these findings to ABL2-rearranged ALL, showing that SH3 domain integrity is required for effective STAMP inhibitor binding and signaling inhibition, with asciminib achieving in vivo efficacy comparable to dasatinib only when this structural prerequisite is met [28]. Although our cases involved classical *BCR::ABL1*-positive disease rather than ABL-class fusions, these data provide a mechanistic framework to explain heterogeneous responses to asciminib monotherapy and underscore the importance of combination strategies in biologically complex or heterogeneous disease. Case 3 underscores the limitations of asciminib in the setting of profound immunosuppression, multiorgan failure, and overwhelming infection. While asciminib contributed to disease control earlier in the disease course, it could not overcome advanced clinical fragility. This mirrors real-world data showing diminished benefit when asciminib is introduced late, reinforcing the need for timely integration before irreversible decline.

The inclusion of Case 3 is vital for illustrating the real-world boundaries of targeted therapies in advanced disease. While the patient ultimately succumbed to refractory multiorgan failure driven by overwhelming infection, this fatal outcome was a consequence of severe clinical complications and profound immunosuppression, not an inherent failure of asciminib’s anti-leukemic activity. In fact, asciminib successfully contributed to MRD-negative remission earlier in his disease course. Case 3 mirrors real-world data showing diminished overall benefit when novel agents are introduced late in the context of clinical fragility, strongly reinforcing the scientific rationale for the timely integration of asciminib before irreversible decline occurs [17].

## 4. Conclusions

Collectively, these three cases and the entirety of available evidence establish asciminib as a versatile but context-dependent agent in Ph+ ALL. Its greatest clinical value lies in combination regimens, bridging to cellular therapies or transplantation, and maintenance strategies, rather than monotherapy. Emerging structural biomarkers such as SH3-domain retention may further refine patient selection. Prospective trials are now required to define optimal sequencing, combinations, and maintenance strategies, particularly in CNS-involved disease and post-CAR-T cell relapses.

## Figures and Tables

**Table 1:** Clinical characteristics and treatment course of three patients with Philadelphia chromosome-positive acute lymphoblastic leukemia treated with asciminib.

Variable	Case 1	Case 2	Case 3
Age/Sex	29 years/Female	37 years/Male	30 years/Male
Underlying Disease	CML in lymphoid blast crisis	CML transformed to Ph+ pre-B ALL	Ph+ (p190) B-ALL
*BCR:: ABL1* Features	Positive; no kinase mutation	T315I mutation	p190 *BCR:: ABL1*
Prior TKIs	Dasatinib, ponatinib	Imatinib, dasatinib, ponatinib	Dasatinib, ponatinib
Prior Major Therapies	Hyper-CVAD, FLAG-IDA, blinatumomab	Blinatumomab (refractory), inotuzumab	Hyper-CVAD, inotuzumab
CNS Involvement	Recurrent CNS disease	CSF-positive at relapse	No documented CNS leukemia
Indication for Asciminib	Recurrent CNS relapse; molecular persistence	Post-CAR-T control; T315I relapse	Bridge to CAR-T during 4th relapse
Asciminib Dose	Escalated to 200 mg BID	40 mg BID → 200 mg BID	40 mg BID
Combination Therapy	Intrathecal chemotherapy	Mini-CVAD + inotuzumab + blinatumomab ± ponatinib	Ponatinib (held during ICU course)
Response to Asciminib	CSF clearance; sustained molecular remission	CR with MRD at detection limit → transplant	Initial disease control; therapy interrupted
Final Outcome	Alive; molecular remission on maintenance	Alive; molecular remission post- allo-HSCT	Deceased (ARDS, sepsis, multiorgan failure)
Last Follow-up	December 2025	December 2025	July 2025

Abbreviations: ALL, acute lymphoblastic leukemia; allo-HSCT, allogeneic hematopoietic stem cell transplantation; BID, twice daily; CAR-T, chimeric antigen receptor T-cell therapy; CML, chronic myeloid leukemia; CNS, central nervous system; CR, complete remission; MRD, measurable residual disease; Ph+, Philadelphia chromosome-positive; TKI, tyrosine kinase inhibitor.

**Table 2:** Clinical studies and case reports of asciminib in Ph+ acute lymphoblastic leukemia.

Study/Report	Study Type	Patient Population	Asciminib Regimen	Combination Therapy	Key Outcomes
Luskin et al. [19]	Phase I trial	Newly diagnosed Ph+ ALL and CML lymphoid blast crisis (*n* = 24)	80 mg daily (RP2D)	Dasatinib + prednisone	CHR 100% by day 84; CCyR 100%; MRD negativity by flow 89%; *BCR::ABL1* ≤ 0.1% in 74%; favorable safety
Mauro et al. [13]	Phase I	R/R Ph+ ALL (*n* = 28), heavily pretreated	Up to 280 mg BID (RDE 200 mg BID)	None (monotherapy)	Molecular disease stabilization; *BCR::ABL1* ≤ 0.1% in 54%; activity limited by short durability
Chanut et al. [17]	Retrospective	R/R Ph+ ALL and CML-LBC (*n* ≈ 40)	Mainly 200 mg BID	±chemotherapy, TKI, immunotherapy	ORR ~80%; MRD negativity ~55%; improved EFS with combinations; effective bridge to allo-HCT
Zerbit et al. [14]	Case report	R/R Ph+ ALL	40 mg BID	Ponatinib	Durable hematologic and molecular disease control (>12 months); good tolerability
Muhammad et al. [15]	Case report	R/R Ph+ ALL with CNS/extramedullary disease	40 mg BID	Ponatinib; brexu-cel CAR-T	Effective bridge to CAR-T; sustained post-CAR-T remission on maintenance
Strumilowska et al. [16]	Case report	Elderly, multi-relapsed Ph+ ALL	80 mg daily	Inotuzumab	MRD-negative CR after 2 cycles; maintained on asciminib monotherapy
Kumar et al. [18]	Case series (*n* = 3)	Ponatinib-refractory/intolerant Ph+ ALL or CML-LBC	200 mg BID	Mini-hyper-CVD + inotuzumab (±blinatumomab)	All achieved CR; MRD negativity in most; successful bridge to allo-HCT

Abbreviations: ALL, acute lymphoblastic leukemia; allo-HCT, allogeneic hematopoietic cell transplantation; *BCR::ABL1*, breakpoint cluster region—Abelson 1 fusion gene; BID, twice daily; CAR-T, chimeric antigen receptor T-cell therapy; CCyR, complete cytogenetic remission; CHR, complete hematologic remission; CML, chronic myeloid leukemia; CR, complete remission; EFS, event-free survival; LBC, lymphoid blast crisis; MRD, measurable residual disease; ORR, overall response rate; Ph+, Philadelphia chromosome-positive; R/R, relapsed or refractory; RP2D, recommended phase II dose; TKI, tyrosine kinase inhibitor.

**Table 3:** Clinical trials of asciminib in Philadelphia chromosome-positive acute lymphoblastic leukemia.

NCT Number	Phase	Study Status	Study Design/Regimen	Patient Population	Treatment Setting/Objective
NCT06308588	I/II	Recruiting	Asciminib + blinatumomab	Adult Ph+ ALL	Chemotherapy-free combination for R/R disease; assess safety, MRD clearance
NCT07250087	II	Recruiting	Asciminib monotherapy	Adult Ph+ ALL post-alloHCT or CAR-T	Maintenance therapy to prevent post-cellular therapy relapse
NCT02081378	I	Completed	Asciminib monotherapy (dose escalation)	CML or Ph+ ALL	Determine MTD/RDE, safety, PK, preliminary efficacy
NCT06773936	II	Not yet recruiting	Asciminib added to standard frontline therapy	Newly diagnosed adult Ph+ ALL	Intensification of frontline therapy
NCT03595917	I/II	Recruiting	Asciminib + dasatinib + prednisone ± blinatumomab	Ph+ B-ALL or CML lymphoid blast crisis	Dual *BCR::ABL1* inhibition with immunotherapy; frontline or early salvage
NCT07040982	II	Not yet recruiting	Asciminib monotherapy	Adult Ph+ ALL post-CAR-T or alloHCT	Maintenance after cellular therapies

Abbreviations: ALL, acute lymphoblastic leukemia; alloHCT, allogeneic hematopoietic cell transplantation; B-ALL, B-cell acute lymphoblastic leukemia; *BCR::ABL1*, breakpoint cluster region—Abelson 1 fusion gene; CAR-T, chimeric antigen receptor T-cell therapy; CML, chronic myeloid leukemia; MRD, measurable residual disease; Ph+, Philadelphia chromosome-positive; PK, pharmacokinetics; R/R, relapsed or refractory; RDE, recommended dose for expansion.

## Data Availability

All relevant data supporting the findings of this study are included within the article. No additional datasets were generated or analyzed.
